# Correlation between functional drainage and survival in malignant biliary obstruction after percutaneous biliary drainage

**DOI:** 10.1016/j.heliyon.2024.e24088

**Published:** 2024-01-08

**Authors:** Hongzhi Yang, Qiujian Qin, yulin Tang, Wenliang Zhu

**Affiliations:** a Department of General Surgery, Minzu Hospital of Guangxi Zhuang Autonomous Region, No 232 Mingxiudong Road, Nanning 531200, China; b General Surgery, People's Hospital of Laibin, No 159 PanGu Road, Laibin, Guangxi, 546100, China; c Department of Minimally Invasive & Interventional Radiology, Guangxi Medical University Cancer Hospital, No 71 Hedi Road, Nanning 531200, China

**Keywords:** Malignant obstructive jaundice, Percutaneous transhepatic biliary drainage, Advanced cancer, Survival

## Abstract

**Purpose:**

Malignant biliary obstruction (MBO) is common in patients with advanced malignant tumors, leading to poor prognosis and hindering antitumor therapy. The purpose of our study was to assess the survival outcomes for patients under therapy after percutaneous transhepatic biliary drainage (PTBD) and identify prognostic factors associated with survival in patients with MBO.

**Methods:**

From July 2010 to February 2021, 269 patients with MBO secondary to malignant tumor were divided into two groups (functional success and non-functional success). Survival time and prognostic factors were analyzed by Kaplan–Meier curves and the Cox model.

**Results:**

The overall median survival time after PTBD was 4.6 months (95 % IC:3.9–5.3). The 3- and 6-month survival rates were 68.0 % and 38.7 %, respectively. The median survival improved from 3.2 months to 8.4 months when the procedure achieved functional success. Multivariate analysis demonstrated that functionally successful drainage and antitumor treatment after PTBD were independent positive prognostic factors, but the total bilirubin after drainage and tumor size were independent negative predictive values.

**Conclusions:**

Functionally successful drainage could prolong survival time in patients with malignant biliary obstruction. Palliative care after drainage can prolong patient survival and improve their quality of life.

## Highlights

• A successful biliary drainage leads to improved survival in patients with malignant biliary obstruction.
• Palliative care - chemo, radiotherapy, TACE after successful biliary drainage can prolong patient survival and improve their quality of life.
• The evaluation of the decrease in TBIL after biliary drainage and its effect on survival.

## 1 Introduction

Malignant biliary obstruction (MBO) is a severe complication of advanced malignant tumors caused by local invasion or compression of the intrahepatic or extrahepatic bile ducts [[1], [2], [3]]. Jaundice is MBO's most typical clinical manifestation, which is usually found in patients with hepatobiliary cancer, pancreatic cancer, and lymph node metastases of other tumors such as gastric and colon cancer [[4], [5], [6]]. MBO markedly affects the quality of life of patients, causing a loss of appetite, very symptomatic pruritus, steatorrhea, and even cachexia [7]. Persistent or advanced biliary obstruction causes liver dysfunction and even biliary cirrhosis, which ultimately makes it too risky or even impossible to perform surgical treatment and chemotherapy [8]. Thus, interventional treatment of MBO is an important part of the treatment of advanced tumors. Achieving effective and durable biliary decompression is the mainstay of treatment for MBO, including endoscopic retrograde cholangiopancreatography (ERCP), percutaneous transhepatic biliary drainage (PTBD), and endoscopic ultrasound-guided biliary drainage (EUS-BD). Clinically successful drainage with a decrease in serum bilirubin has significantly impacted patient prognosis and survival [2,9]. PTBD is a standard treatment method for patients with unsuccessful or infeasible ERCP. It has the advantages of minor trauma, rapid recovery, and good efficacy. However, the current research data on PTBD in MBO are few, and it is difficult to provide reliable clinical references. The objective of this study was to assess the survival outcomes for patients under therapy after PTBD and identify prognostic factors associated with survival in patients with MBO.

## 2 Materials and methods

### 2.1 Patient selection

From July 2010 to February 2021, the outcomes of patients with MBO treated by PTBD were reviewed retrospectively. This study was approved by the Institutional Review Board (B-2022-386-01) and obtained the informed exemption. The inclusion criteria were as follows: 1) patient with an advanced malignant neoplasm underwent PTBD procedure for the first time; 2) biliary dilation diagnosed by computed tomography (CT) or magnetic resonance cholangiopancreatography (MRCP); 3) laboratory evaluation showing increased serum levels of total and direct bilirubin; 4) Eastern Cooperative Oncology Group (ECOG) score ≤2. The exclusion criteria were as follows: 1) patients with severe coagulation dysfunction; 2) patients with multiple organ failure and unable to undergo PTBD; 3) patients with severe infection; 4) patients lacking procedural information. The collected variables included patient characteristics and history, laboratory data, pathologic type, complications, follow-up treatment, and overall survival.

### 2.2 Definitions

Technical success was defined as successful drainage catheter placement or stent in a biliary stricture. According to the previous study [3,10], functional success was defined as the total serum bilirubin level decreasing to a normal level or less than half of the value before PTBD. All patients were divided into non-functional success and functional success groups. Common adverse reactions were based on the Common Terminology Criteria for Adverse Events (CTCAE) Version 4.0.3 [11]. Complications were classified as major and minor following the reporting standards of the Society of Interventional Radiology [12]. Postprocedural bleeding was defined as a decrease in hemoglobin of >2 g/dl with symptoms of hematemesis or melena after procedure. Biliary tract infection was defined as postprocedural fever >38.5 °C or leukocytosis >10 × 10^9^/L and excluded the infections caused by other etiologies.

### 2.3 Follow-up

The follow-up period was defined as the time from the procedure to the time of death, the end of the study (February 2021) or loss. Follow-up data included patient survival, laboratory data 2 weeks after the procedure, and all follow-up treatment. Overall survival (OS)was the time from the start of PTBD to the patient's death or the end of follow-up.

### 2.4 Statistical analysis

Statistical analysis was performed using SPSS v22.0 (IBM Corp, Armonk, NY). Continuous variables were presented as mean ± standard deviation, and categorical variables are described by frequency and percentage. Quantitative data with a normal distribution were analyzed using the *t*-test; otherwise, they were analyzed by Mann–Whitney *U* test. Categorical variables were analyzed by the chi-square test or Mann–Whitney *U* test. The Cox model was used to analyze the prognostic factors, and survival time was calculated using Kaplan–Meier curves and the Log-rank tests. For all analyses, p-values <0.05 were considered statistically significant.

## 3 Results

### 3.1 Patient characteristics

A total of 269 patients, including 179 females and 90 males, with MBO were included in this study. The median age of the patients was 58 ± 12 years. The sites of the primary neoplasm were liver cancer (n = 44, 16.4 %), cholangiocarcinoma (n = 74, 27.5 %), periampullary duodenal cancer (n = 68, 25.3 %), pancreatic cancer (n = 14, 5.2 %), and colon cancer (n = 54, 20.1 %). The mean diameter of the tumor at the site of biliary obstruction was 5.2 ± 0.2 cm. 89 patients underwent resection of the primary neoplasm. The location of MBO was the hepatic hilar area in 161 patients and the common bile duct in 108. A total of 109 patients underwent anti-tumor treatment after bile duct drainage, including 92 patients who received systemic chemotherapy and 17 patients who received local minimally invasive interventional therapy for the metastatic or primary tumor. The technical success rate was achieved in 260 patients (96.7 %), among whom 8 underwent PTBD after the failure of ERCP, and six underwent ERCP after the failure of PTBD. Unfortunately, due to less detailed stent implantation records and the small number of stent-related cases in the past, we did not analyze stent-related factors. The clinical characteristics of the patients are summarized in Table 1.

**Table 1:** Characteristics of the 269 patients with MBO underwent PTBD.

Variables	Total (*n* = 269)
Age (years)	58 ± 12
Sex	
Male	90 (33.5 %)
Female	179 (66.5 %)
Primary tumor	
Hepatocellular	44 (16.4 %)
Cholangiocarcinoma	74 (27.5 %)
Gastric	68 (25.3 %)
Pancreas	14 (5.2 %)
Colorectal	54 (20.1 %)
Others	15 (5.6 %)
Diameter (cm)	5.2 ± 0.2
Previous surgery (primary)	
No	180 (67 %)
Yes	89 (33 %)
Cause of obstruction	
Invasion	48 (17.8 %)
Compression	221 (82.2 %)
Site of biliary stenosis	
Hilum	161 (60 %)
Common bile duct	108 (40 %)
Post-drainage treatment	
Yes	109 (41 %)
No	160 (60 %)
WBC (cells × 10^9^/L)	7.60 ± 3.23
Hemoglobin (g/L)	110.7 ± 18.7
Complications	
Cholangitis	77 (29 %)
Hemorrhage	3 (1 %)

Note: Continuous variables are expressed as mean ± standard error. Categorical variables are expressed as n (%). PTBD: Percutaneous transhepatic biliary drainage, WBC: White blood cell, MBO: Malignant biliary obstruction.

### 3.2 Changes in laboratory data before vs. after biliary drainage

The average pre-procedural serum total bilirubin level was 248 ± 130 μmol/L, which decreased to 158 ± 134 μmol/L after PTBD. The laboratory evaluation of liver function (including total bilirubin, direct bilirubin, aspartate aminotransferase (AST), alanine transaminase (ALT)) decreased significantly after PTBD compared to before (paired-sample *t*-test, *p* < 0.05). Changes in laboratory data before and after biliary drainage are shown in (Table 2).

**Table 2:** Changes in laboratory data before vs. after biliary drainage (using the paired-sample *t*-test).

	Before biliary drainage	After biliary drainage	*P-value*
ALT (U/L)	129 ± 180	67 ± 57	<0.001
AST (U/L)	124 ± 92	85 ± 148	<0.001
Direct bilirubin (μmol/L)	204 ± 105	131 ± 108	<0.001
Total bilirubin (μmol/L)	248 ± 130	158 ± 134	<0.001
ALB (g/L)	35.65 ± 11.1	36.8 ± 7.1	0.859
ALBI score	−1.50	−1.70	0.002

Note: Data are presented as the mean ± standard deviation. ALT: Alanine aminotransferase, AST: Aspartate aminotransferase, ALB: albumin, ALBI: albumin-bilirubin. *P*-values <0.05 were considered statistically significant.

### 3.3 Changes in ALBI grade before vs. after biliary drainage

The albumin-bilirubin (ALBI) grade was also improved after procedure (*p* = 0.001), imparting clinical and statistical significance (Table 3).

**Table 3:** Changes in ALBI grade before vs. after biliary drainage (using chi-square or Fisher's exact test).

ALBI grade	Before biliary drainage	After biliary drainage	*P*-value
I	6	23	0.001
II	135	146	
III	128	100	

Note: ALBI: Albumin-bilirubin, *P*-values <0.05 were considered statistically significant.

### 3.4 Overall survival

**Fig. 1:**
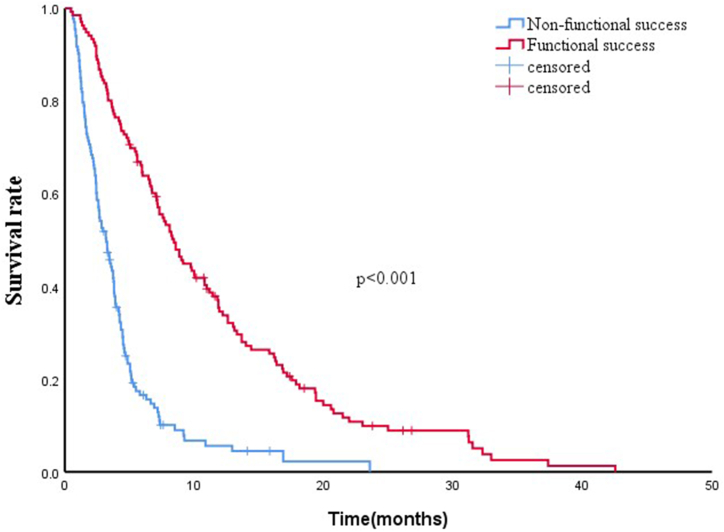
Comparison of the overall survival between functional success group and non-functional success groups.

**Fig. 2:**
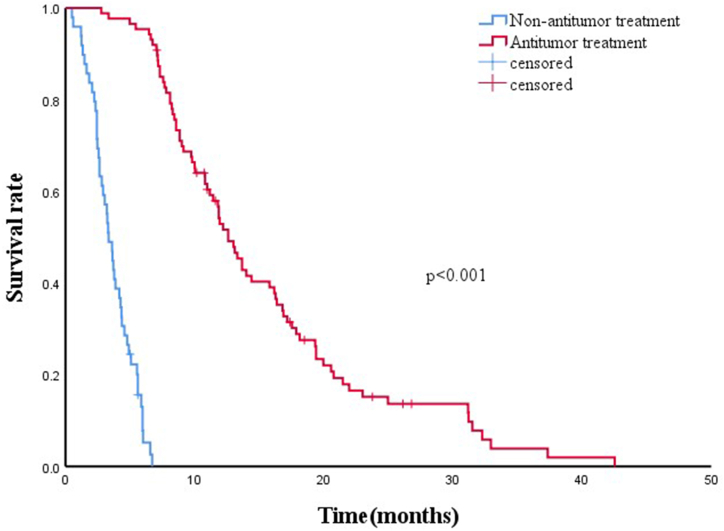
Comparison of the overall survival between antitumor treatment group and non-antitumor treatment group.

The median OS was 4.6 months (95 % CI:3.9–5.3). The 3- and 6-month survival rates were 68.0 % and 38.7 %, respectively. Kaplan–Meier curves and Log-rank tests showed that the median OS of the functionally successful group (n = 136) was significantly better than that of the non-functionally successful group (n = 133) (8.4 months *vs.*3.2 months; *p* < 0.001; Fig. 1). The functionally successful group were divided into two groups with antitumor treatment (n = 87) and non-antitumor treatment (n = 49). The baseline did not reveal any significant differences between the two groups (Table 4). The median OS of antitumor treatment group, in which the patients were treated with chemotherapy, transarterial chemoembolization (TACE), or radiofrequency ablation, was significantly better than that of the non-antitumor treatment group (12.6 months, 95 % CI:11.2–14) vs. 3.3 months, 95 % CI: 2.7–3.9; p < 0.001; Fig. 2).

**Table 4:** Comparison of baseline between antitumor treatment group and non-antitumor treatment group (Mann-Whitney *U* test/χ2 test).

Variables	antitumor treatment (87)	non-antitumor treatment (49)	P value
Age (years)	57 ± 11	57 ± 12	0.992
Sex			
Male	60	34	0.489
Female	32	24	
Primary tumor			0.657
Hepatocellular	13	11	
Cholangiocarcinoma	34	14	
Gastric	24	18	
The pancreas	4	3	
Colorectal	12	7	
Others	5	5	
Diameter (cm)	4.7 ± 2.7	5.1 ± 2.4	0.335
Previous surgery (primary)			0.598
No	59	40	
Yes	33	18	
Cause of obstruction			1.0
Invasion	77	48	
Compression	15	10	
Site of biliary stenosis			0.737
Hilum	51	30	
Common bile duct	41	28	
WBC (cells × 103/μL)	7.3 ± 3.4	7.9 ± 2.9	0.488
Hemoglobin (g/L)	112.8 ± 16.1	112.4 ± 19.5	0.901
A decreased proportion of total serum bilirubin	0.66 ± 0.15	0.65 ± 0.12	
Pre-DBIL (μmol/L)	189 ± 97	219 ± 108	0.327
Pre-TBIL (μmol/L)	229 ± 120	266.9 ± 129.3	0.441
Post-DBIL (μmol/L)	60.5 ± 50.8	77.5 ± 44.7	0.458
Post-TBIL (μmol/L)	76.1 ± 56.5	90.8 ± 50.2	0.595

Note: Continuous variables are expressed as mean ± standard error. Categorical variables are expressed as n. WBC: White blood cell, Pre-DBIL: Direct bilirubin before biliary drainage, Pre-TBIL: Total bilirubin before biliary drainage, Post-DBIL: Direct bilirubin after biliary drainage, Post-TBIL: Total bilirubin after biliary drainage.

Univariate analysis showed that a hemoglobin <90 g/L (moderate anemia or less), abnormally increased liver dysfunctional data (total bilirubin, direct bilirubin, AST), larger tumors, and an ALBI grade > −1.39 were poor prognosis factors. Functionally successful drainage and antitumor treatment improved survival. Multivariate analysis demonstrated that functionally successful drainage and antitumor treatment were independent positive prognostic factors, but post-TBIL and tumor size were independent negative prognostic factors (Table 5).

**Table 5:** Results of univariate and multivariate analysis to identify independent prognostic factors for overall survival (using Cox regression model) after biliary drainage.

Variables	HR	95 % IC	P	HR	95 % IC	P
Sex (female)	1.14	0.88–1.47	0.318			
Age	1.00	0.99–1.01	0.746			
Site of primary neoplasm (Hepatocellular)			0.053			
Previous surgery (primary)	1.179	0.913–1.523	0.207			
Infection (YES)	1.258	0.964–1.642	0.091			
HGB (<90 g/L)	1.947	1.341–2.828	0.001			
Pre-ALT (U/L)	1.000	0.999–1.001	0.386			
Pre-AST (U/L)	1.000	0.999–1.001	0.848			
Albumin (g/L)	0.988	0.973–1.004	0.132			
Pre-DBIL (μmol/L)	1.001	1.000–1.003	0.022			
Pre-TBIL (μmol/L)	1.001	1.000–1.002	0.016			
Ratio of pre-ALBI			0.006			
≤ −1.39						
> −1.39	1.411	1.104–1.802				
Post-ALT (U/L)	1.001	0.999–1.003	0.35			
Post-AST (U/L)	1.003	1.002–1.004	0.001			
Post-DBIL (μmol/L)	1.005	1.003–1.006	0.001			
Post-TBIL (μmol/L)	1.004	1.003–1.004	0.001	1.001	1.000–1.003	0.018
Ratio of Post-ALBI			0.001			
≤ −1.39						
> −1.39	2.035	1.575–2.630				
Diameter (cm)	1.073	1.030–1.118	0.001	1.072	1.026–1.120	0.002
Obstruction level						
Hilar	0.87	0.68–1.11	0.27			
Common bile duct						
Functional success			0.001	0.683	0.484–0.963	0.030
No						
Yes	0.293	0.224–0.382				
non-antitumor treatment			0.001	0.044	0.025–0.077	0.001
No						
Yes	0.034	0.020–0.059				

Note: HR: Hazard ratio, CI: Confidence interval. Pre-DBIL: Direct bilirubin before biliary drainage, Pre-TBIL: Total bilirubin before biliary drainage, Post-DBIL: Direct bilirubin after biliary drainage, Post-TBIL: Total bilirubin after biliary drainage.

Complications after biliary drainage included mainly bleeding and fever. Post-drainage bleeding occurred in three patients and stopped after giving fresh frozen plasma or correcting the coagulopathy. Seventy-seven patients developed postoperative fever or an increased proportion of the total leukocyte count. After 3–5 days of antibiotic treatment, the body temperature and level of leukocytes returned to normal.

## 4 Discussion

MBO often occurs in the advanced stage of locally invasive, malignant neoplasms, which directly invade or compress the biliary tree either by the primary tumor or via nodal metastases. The resultant obstructive jaundice further leads to the failure of liver function. This study aimed to investigate the prognostic factors of MBO and the effect of drainage and antitumor treatment on survival time.

In our study, while the technical success rate was 96.7 %, which was similar to some previous findings, the clinical success rate of only 50.6 %, as defined by a decrease to normal or a >50 % decrease, was less than that observed in the study of Zhang et al. (76.5 %) [13]. In the Zhang study, however, clinical success was defined as a 20 % decrease in serum bilirubin, which was considerably less than our definition (50 %). Moreover, our relatively lower clinical success rate may be explained: the baseline bilirubin level in our study was relatively higher; included patients were advanced cancer, and 161 had hilar invasion, which was considered a high failure factor of biliary drainage.

The ALBI grade was statistically significant, with a higher grade suggesting a worse prognosis. In another study of patients with hepatocellular cancer, the Child-Pugh class was included and showed that Child-Pugh C liver function was a factor affecting survival [3,14]; however, there was no comparison of the effect of two liver function classifications on survival time in obstructive jaundice. In our study, multivariate statistics showed that after biliary drainage, the resultant total bilirubin was an independent prognostic factor for poor survival although the pre-drainage serum bilirubin levels were generally not associated with clinical success, similar to the findings of a previous study [13,15].

Our study and several others have demonstrated that successful drainage of obstructive jaundice followed by the ability to provide local therapy, such as TACE, chemotherapy, radiotherapy, or radiofrequency or systemic chemotherapy, significantly prolongs survival time and improves the quality of life of patients [2,[16], [17], [18]]. The results of our multivariate analysis showed that functionally success drainage and antitumor treatment were independent factors of better survival and improved patient outcomes in these patients with advanced tumors. The median OS in this study was 135 days, which was slightly greater than the intervals of 79–104 days reported in previous studies [16,[18], [19], [20]]. What is important is that the median survival time of 8.4 months in the patients with functionally successful drainage was significantly greater than in the non-functionally successful group (3.2 months). In the functionally successful group, patients who received chemotherapy, radiofrequency ablation, or TACE had a greater survival rate than those with non-antitumor treatment. Although other studies of patients with colorectal cancer [[19], [20], [21]] have not shown a relationship between biliary drainage and survival, such differing results might be related to different prognostic factors and patient characteristics. However, our study demonstrated that survival was closely associated with functionally successful drainage and anti-tumor treatment. However, different studies might generate different results due to different prognostic factors and patient characteristics.

As in multiple other studies, common complications of biliary drainage include cholangitis, hemorrhage, pancreatitis, pleural injury, biliary-heart reflex, and displacement of the biliary drainage tube after PTBD. Among them, infection and hemorrhage are the main causes of PTBD-related death. In our study, although postoperative infection occurred in 28 % of patients, body temperature and leukocytes returned to normal after antibiotic treatment. No patient died due to infection, and the 30-day mortality rate was 5 %, compared to 2%–19.8 % in previous studies [[22], [23], [24]].

Our study has several limitations that warrant discussion. First, this study was retrospective and included multiple sites of the primary neoplasm, with the expected heterogeneity in response to treatment. Second, no subgroup analysis of stenting alone was performed due to the small number of stent-related cases. Third, we only evaluated the outcomes of PTBD patients and did not compare those patients who underwent the less invasive means of internal biliary drainage like ERCP/endoprosthesis. Fourth, because clinical symptoms such as itching, weakness, nausea, and nutritional status were excluded from our study, we did not analyze the patients' quality of life. Fifth, no data on general PS were analyzed in this study, which may lead to selection bias between groups. This imbalance of clinical background may have influenced the difference in survival between functional and non-functional success groups. Therefore, randomized controlled trials are needed to support our findings possibly focusing on specific patient groups like hepatic cancer, cholangiocarcinoma, periampullary and pancreatic cancers, and metastases from colon cancer.

In conclusion, functional drainage of PTBD and antitumor treatment after PTBD can prolong MBO patients' survival.

## Funding statement

This study has received funding by Natural Science Foundation of Guangxi (fund No: Z20210846).

## Data availability statement

Data will be made available on request.

## Ethics approval and consent to participate

This retrospective study was performed in accordance with the Declaration of Helsinki of the World Medical Association. It was waived by Institutional Review Board of the Guangxi medical cancer hospital, and the informed consent of patients was waived because of its low risk.

## Additional information

No additional information is available for this paper.

## CRediT authorship contribution statement

**Hongzhi Yang:** Conceptualization, Writing – original draft. **Qiujian Qin:** Data curation, Methodology. **yulin Tang:** Data curation, Formal analysis, Validation. **Wenliang Zhu:** Supervision, Writing – original draft, Writing – review & editing.

## Declaration of competing interest

The authors declare that they have no known competing financial interests or personal relationships that could have appeared to influence the work reported in this paper.
